# O-10 - EVALUATING THE NATIONAL IMPLEMENTATION AND IMPACT OF TARGET ANTIMICROBIAL STEWARDSHIP TRAINING IN ENGLAND

**DOI:** 10.1093/pubmed/fdag046.010

**Published:** 2026-07-09

**Authors:** Ms Ming Xuan Lee, Ms Jade Meadows, Dr Nina Zhu, Dr Raheelah Ahmad, Dr Helena Wehling, Mrs Jo Taylor-Egbeyemi, Dr Louise E Smith, Dr Dale Weston, Dr Kieran Hand, Dr Donna M Lecky

**Affiliations:** Primary Care and Interventions Unit, UK Health Security Agency; Primary Care and Interventions Unit, UK Health Security Agency; Centre of Antimicrobial Optimisation, Imperial College London; School of Health and Medical Sciences, City St George's University of London; School of Health and Medical Sciences, City St George's University of London; Behavioural Science and Insights Unit, UK Health Security Agency; Behavioural Science and Insights Unit, UK Health Security Agency; Behavioural Science and Insights Unit, UK Health Security Agency; Behavioural Science and Insights Unit, UK Health Security Agency; AMR Programme, Medical Directorate, NHS England; Primary Care and Interventions Unit, UK Health Security Agency

## Abstract

**Background:**

Evidence suggests that 20-30% of antibiotics prescribed in primary care are unnecessary highlighting the need for effective antimicrobial stewardship (AMS). The TARGET toolkit provides evidence-based resources and AMS training support positive prescribing behaviour. In October 2022, UKHSA and NHSE initiated a national cascade rollout via regional leads, Integrated Care Boards and primary care teams. A multicomponent evaluation was conducted to understand implementation readiness, delivery processes, and impact on prescribing.

**Objectives:**

To inform effective implementation, assess reach and fidelity, and evaluate the effect of the TARGET training on antibiotic dispensing in England.

**Methods:**

Implementation evaluation: Two stakeholder workshops (n=22; n=14) explored anticipated and experienced implementation challenges using the Theoretical Domains and ERIC framework models.

Process evaluation: We assessed reach through survey data collection and obtained qualitative feedback from regional representatives (n=22), six-month follow up data (n=67), and participant interviews (n=8).

Impact evaluation: Analysis of monthly antibiotic dispensing using interrupted time-series analysis and difference-in-differences modelling across ICBs that adopted training.

**Results:**

Implementation evaluation: Identified barriers included limited capacity, time, varying confidence in delivering training, competing priorities, and difficulty evidencing short term AMR impact. Enablers included regional champions, alignment with professional development, promotion of benefits, and financial incentives.

Process evaluation: From October 2022 to March 2025, ≥2,455 clinicians were trained, with high intention to change behaviour: 94% planned to discuss AMS at practice meetings; 90% intended to embed a whole practice approach. At six months, 94% (n=61/65) had implemented planned actions; 97% (n= 31/32) reported increased confidence.

Impact: Impact analysis showed adopting ICBs dispensed 0.9 fewer antibiotic prescription items per 1,000 patients in the first month following roll out, with an additional monthly reduction of 0.2 items.

**Conclusions:**

National implementation of TARGET AMS training is feasible and effective, but requires regional flexibility, ongoing support, and sustained follow-up to optimise long-term stewardship outcomes.

